# Predicting the optoelectronic properties of nanowire films based on control of length polydispersity

**DOI:** 10.1038/srep25365

**Published:** 2016-05-09

**Authors:** Matthew J. Large, Jake Burn, Alice A. King, Sean P. Ogilvie, Izabela Jurewicz, Alan B. Dalton

**Affiliations:** 1 University of Surrey, Department of Physics, Guildford, GU2 7XH, United Kingdom; 2 University of Sussex, Department of Physics, Brighton, BN1 9RH, United Kingdom

## Abstract

We demonstrate that the optoelectronic properties of percolating thin films of silver nanowires (AgNWs) are predominantly dependent upon the length distribution of the constituent AgNWs. A generalized expression is derived to describe the dependence of both sheet resistance and optical transmission on this distribution. We experimentally validate the relationship using ultrasonication to controllably vary the length distribution. These results have major implications where nanowire-based films are a desirable material for transparent conductor applications; in particular when application-specific performance criteria must be met. It is of particular interest to have a simple method to generalize the properties of bulk films from an understanding of the base material, as this will speed up the optimisation process. It is anticipated that these results may aid in the adoption of nanowire films in industry, for applications such as touch sensors or photovoltaic electrode structures.

Indium Tin Oxide (ITO) is a widely used transparent conductive oxide with applications as a transparent electrode material in the vast majority of electronic devices available today. Since the first widespread adoption of touch-screen devices shortly after the turn of the millennium, the price of bulk Indium has increased significantly. Supply uncertainty has also applied pressure to the ITO industry, leading to investigation of alternative materials over the last decade or so. Furthermore, looking toward next generation devices which are anticipated to be curved or flexible, ITO is deemed to be unsuitable due to its brittle crystalline structure and high deposition temperature.

Alternative materials investigated as ITO replacements have included graphene, carbon nanotubes, metal mesh and random metal nanowire films. Of these, silver nanowire (AgNW) films have emerged as the strongest competitor, due to transmittances and conductivities which can match and readily exceed those of ITO.

The rapid adoption of nanowire films in transparent conductor applications requires a robust method for designing materials capable of meeting application-specific performance requirements. These requirements typically include high transmittance and low sheet resistance. Also of importance to displays and sensor applications is low haze (the ratio of diffuse to specular transmittance). Higher haze is a useful feature in photovoltaic applications, where this improves coupling and trapping of light inside the device, leading to increases in device efficiency.

Typically a mathematical expression relating film transmittance T and sheet resistance 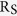 (termed a T-R expression) is used to gauge the relative performance of the transparent electrode. Two established forms for this relation exist at present; the first describes continuous bulk films^1^, and the second describes percolating nanostructured materials^2^;


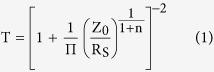


where 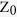 is the impedance of free space (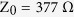); 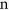 is a percolative exponent describing the power law relation between 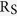 and the film thickness; 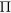 is a percolative figure of merit, where higher values couple low values of 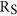 to high values of 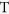.

Prior work presented in the literature has recognised a qualitative relationship between the mean length of silver nanowires and their T-R performance^3^,^4^,^5^,^6^,^7^.

Ultrasonication has been demonstrated as a technique capable of manipulating the length distribution of high aspect ratio nanoparticles^8^,^9^. The application of high pressure amplitude acoustic waves to a nanoparticle dispersion induces the formation and growth of cavitation bubbles. These bubbles can be stable, oscillating in phase with the driving acoustic field, or collapse chaotically generating large local temperature increases and local fluid flow rates. It is the latter behavior, known as inertial cavitation, which leads to the process of ultrasonic scission of rod-like nanoparticles^9^.

## Results

**Figure 1:**
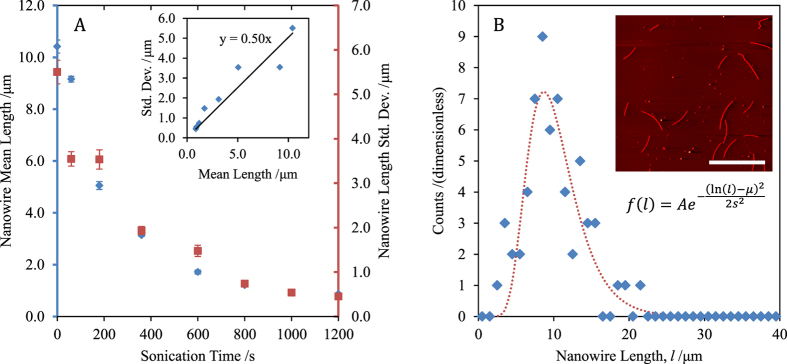
(**A**) Shows how the measured length statistics 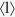 (mean length) and 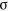 (standard deviation) vary with sonication time. A Fisherbrand FB15051 bath sonicator with nominal 320 W peak output in swept-frequency mode was used. The inset illustrates the approximate relationship between 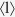 and 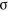. (**B**) Shows a typical length distribution measured from AFM data (a characteristic image is shown in the inset; four different fields are used to compile the histogram data for each sample. The scale bar is 30 μm). The fitted distribution function is log-normal, with parameters μ = 2.14 ± 0.01 μm and s = 0.37 ± 0.01 μm which correspond to a mean length of 〈l〉 = 9.2 ± 0.1 μm and standard deviation of σ = 3.5 ± 0.2 μm (fitted parameter variances are evaluated using a delete-1 jackknife resampling method^17^).

Figure 1(A) illustrates the capability of ultrasonication to modify the length distribution of silver nanowires in solution by control of the sonication time. As-received silver nanowire material from a commercial supplier was diluted in IPA to a concentration suitable for spray deposition prior to treatment in a room temperature ultrasonic bath. The initial nanowire population has a diameter of d = 30 ± 4 nm (measured by TEM), a mean length of 〈l〉 = 10.4 μm and a length standard deviation of σ = 5.5 μm (measured by AFM, see Fig. 1(B) for a characteristic measurement). The inset to Fig. 1(A) shows that an approximate relationship is maintained between the standard deviation and mean length.

It has long been understood that the performance of nanowire films as transparent conductors is linked to the length of the nanowires used^3^,^4^,^5^,^6^,^7^. We develop a modification to the theory given by De *et al.*^2^, described in detail in the Supplementary Information, which clarifies the link between nanowire length statistics and the resulting electro-optical film performance.

From continuum percolation theory we have a percolative scaling relation for film conductivity 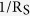 that depends on the nanowire density measured by the filling factor 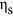;


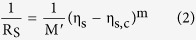


where 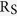 is the sheet resistance; M′ is a material constant; m is the percolative exponent of conductivity (which has a nominally universal value of m = 1.30 in two dimensions). The filling factor is defined as the total squared length of rods per unit area of the substrate. This measure is convenient since the percolation threshold density, 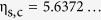
10, is expected to be universal for all rod-like objects and independent of their length distribution. We can relate 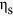 to the area fraction 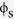 (the total projected area of objects per unit area of the substrate) by 
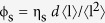
, and by inversion of equation (2) relate the area fraction to the sheet resistance;


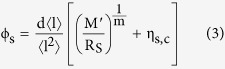


We adopt a semi-empirical model of the film transmission in the form of the Beer-Lambert law, and deduce that;


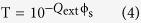


where 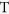 is the film transmittance and 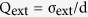 is the dimensionless extinction efficiency of the nanowires (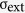 is the extinction cross-section per unit length). Substitution of equation (3) into equation (4) gives a new T-R expression;


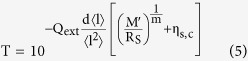


By considering the limit as 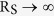 we discover a ‘critical transmittance’ term, representing an upper bound on the transmittance of a conducting film;


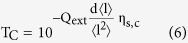


By considering the complementary limit, where 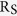 is small such that 
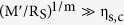
, and comparing the first order expansion of equation (5) to that of equation (1), we arrive at a percolative figure of merit comparable to that derived by De *et al.*^2^ (see Supplementary Information for details);


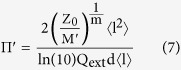


From equation (7) we expect film performance to decrease with decreasing nanowire length (since the term 〈l^2^〉/〈l〉 scales with the mean length). We would also expect that larger diameter nanowires should not perform as well for a given length distribution (bearing in mind that the extinction efficiency 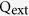 is monotonic with, but not linear in, the nanowire diameter).

**Figure 2:**
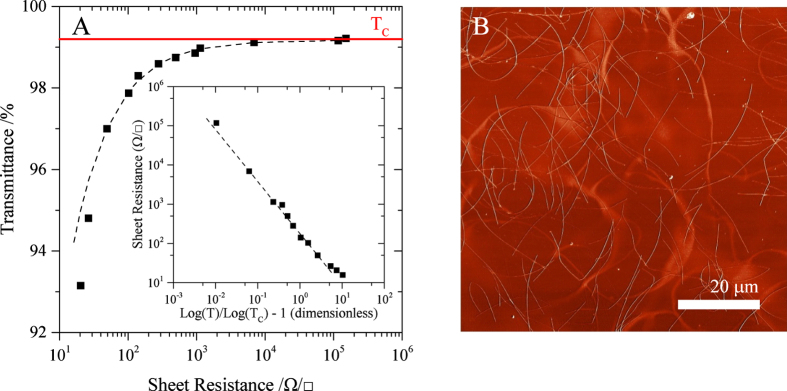
(**A**) shows typical sheet resistance and transmittance data for a film prepared using unsonicated nanowire material. The critical transmittance is calculated to be 99.1%. Equation (5) is fitted to the data, giving fitted values of 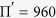, and m = 1.33 ± 0.04; the inset shows the same data on a log-log plot of 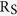 against 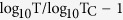 (which corresponds to 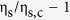). (**B**) shows a phase-contrast AFM image of a representative silver nanowire network with T(550 nm) = 98.6% and 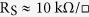, the scale bar is 10 μm.

In order to confirm the predicted effect of length distribution on the performance of nanowire films, sonicated material is spray deposited onto glass substrates, with transmittance and sheet resistance values collected between sequential spray passes. Fig. 2(A) shows a typical T-R data series for unsonicated nanowire material. Equation (5) has been fitted to the data with m and M′ as the only free parameters; the AFM length distribution measurements of Fig. 1 are used in the calculations, along with 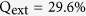 (calculated using Lumerical FDTD^11^ for nanowires of 30 nm diameter).

**Figure 3:**
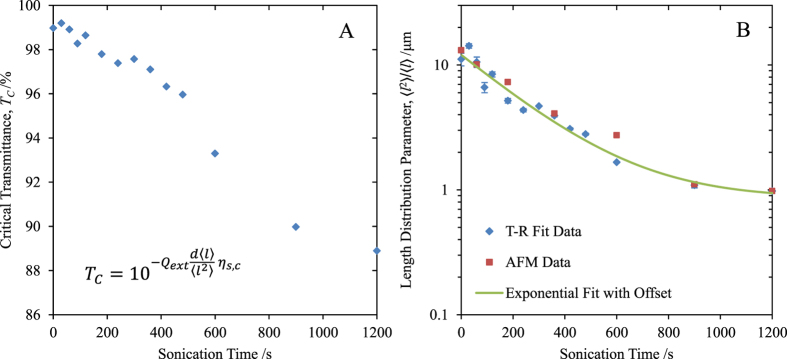
(**A**) Plot of 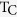 against sonication time for films formed of sonicated material (the average of three samples is shown). The data are derived by fitting equation (5) to T-R data using the variables M′, m and 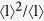 as fitting parameters. (**B**) Plot of the fitted parameter 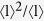 against sonication time. The AFM values are derived from the data presented in Fig. 1(A). The fit to the T-R data results is defined by 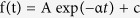, where A = 11.2 μm, α = 0.0040 s^−1^ and c = 0.85 μm, over the 0 to 1200 s range.

By introducing the length statistic 〈l^2^〉/〈l〉 as a third fitting parameter it is possible to identify an aspect of the nanowire length distribution given the T-R data alone (provided the nanowire diameter, and therefore extinction properties, are known). Figure 3(A) shows the fitted value of the critical transmittance for nanowire material sonicated for increasing durations, where each data point is an average over three samples. Fig. 3(B) plots the fitted value of the length distribution parameter, 〈l^2^〉/〈l〉; for comparison the values of 〈l^2^〉/〈l〉 calculated from the AFM measurements of Fig. 1(A) are shown.

Agreement between the T-R fitted values and the AFM measurements is extremely good. We can suggest that the macroscopic behaviour of transparent conductive films formed from nanowires has a robust dependence on the length distribution of those nanowires, and that manipulation of the nanowire length distribution can be achieved through the use of ultrasonication. We can also infer that the described T-R model correctly captures the effect of variation in the length distribution on the critical transmittance.

The trend fitted to the data in Fig. 3(B) indicates that there exists a limiting value of 〈l^2^〉/〈l〉 obtainable using this ultrasonic scission process. This is consistent with the mechanism suggested by Huang^8^, whereby the limiting length 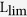 of nanorods with diameter 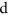 is related to the solvent viscosity 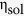, cavitation bubble radius 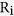 and wall collapse rate 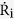, and the tensile strength of the material of interest 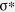 by;


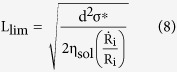


Utilising the approximate cavitation parameters (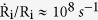)^8^ and the nominal viscosity of our dispersing solvent (IPA) at room temperature (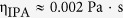 12), and assuming that the length distribution parameters are related as in the inset in Fig. 1(A) (so that 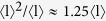), we anticipate that 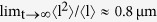 in excellent agreement with the fitted value of 0.85 μm from Fig. 3.

**Figure 4:**
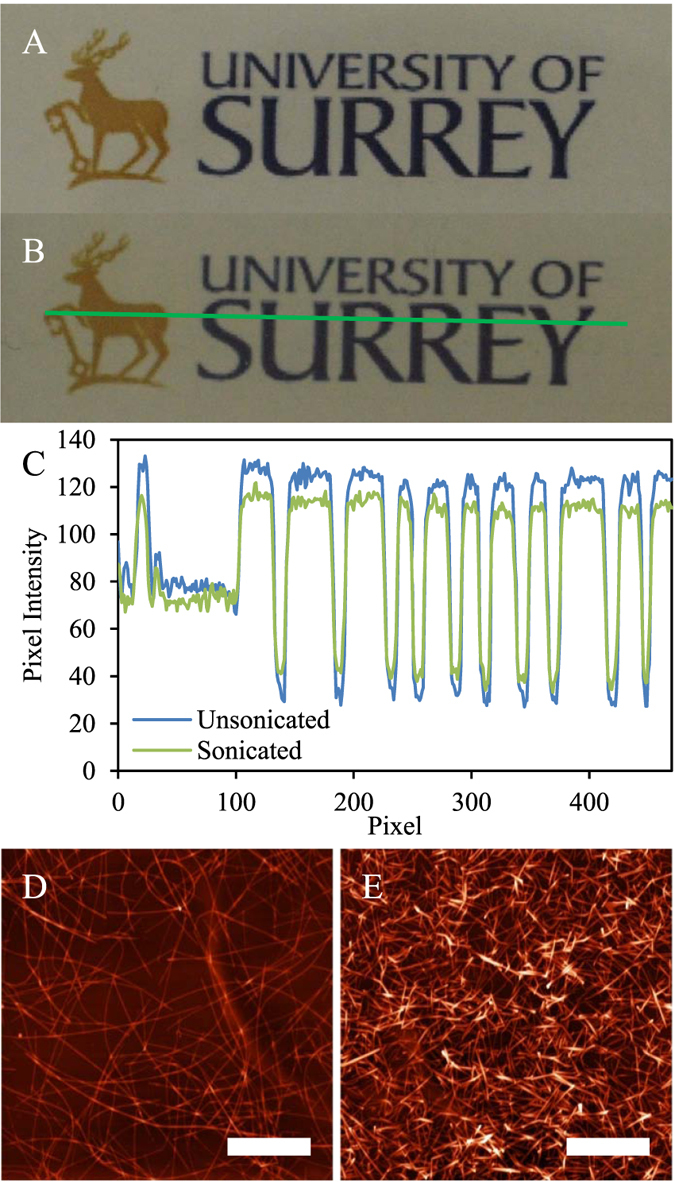
(**A**,**B**) show photographic images of films sprayed using unsonicated (〈l〉 = 12.4 ± 0.1 μm, σ = 4.5 ± 0.1 μm) and sonicated (〈l〉 = 1.54 ± 0.1 μm, σ = 0.89 ± 0.03 μm) nanowires respectively, with a sheet resistance of approximately 60 Ω/□. (**C**) plots the pixel intensities along the line section (green line) shown in Fig. 4(**B**) for both images (**A**,**B**), extracted using ImageJ^16^. Pixel intensities are integers on the range [0,255] representing the equivalent grayscale value at each pixel. (**D**,**E**) show representative AFM topography for the films (**A**,**B**) respectively; the scale bars are 5 μm.

Figure 4 qualitatively illustrates the ability of solution-phase ultrasonication to tailor the electro-optical properties of deposited nanowire films. Figure 4(A) shows a photograph of a film utilizing unsonicated nanowires with mean length 〈l〉 = 12.4 μm, and (B) shows a comparable film produced using sonicated nanowires with mean length 〈l〉 = 1.54 μm. Films (A) and (B) have the same sheet resistance (60 Ω/□), but film (A) has a significantly higher transmittance and lower haze than (B). Both of these points are illustrated by the pixel intensity profile in Fig. 4(C). As can be seen, the contrast between light and dark areas of the image is reduced for the film of Fig. 4(B), indicating higher haze. Also, the average pixel intensity for the film (B) profile is 6% lower than that for film (A).

This type of control may prove useful in situations where optimization of multiple parameters is of interest. For example, sacrificing some transmittance to increase film scattering is a useful feature in photovoltaic transparent electrodes as increased light coupling into, and light trapping within, the device is important^13^. Also, the higher area coverage of nanowires on the surface will enhance the collection efficiency of photo-generated charge carriers at the electrodes.

## Discussion

By evaluating the effect of controlled sonication on the length distribution of AgNWs it is possible to determine a generalized relationship for the macroscale optoelectronic properties of a percolating film derived of such nanowires. We expect that this method can be generalized to any nanowire films for which the nanoscale properties are known, or indeed vice versa. This expression allows for a simple and robust method to determine either film properties from known nanoscale data or the nanoscale properties of a bulk film based on macroscopic T-R measurement series.

The process of ultrasonic scission in this procedure is dependent on nanowire diameter and solvent viscosity^8^, and will likely also be influenced by the dispersion concentration as well as other factors. This gives a broad range of control parameters for optimization of film materials, complementing the use of hybrid-type films in transparent electrode applications^14^,^15^.

## Methods

### Silver nanowire dispersion and ultrasonication

AgNW dispersions were purchased from a commercial supplier, with an as-received silver concentration in the range of 1–5 mg/mL. The material is used as-received and diluted in IPA at a mass ratio of 0.75:10. Diluted AgNW dispersions were ultrasonicated using a Fisher Scientific FB15051 (nominal ~300 W output) ultrasonic bath in ice. After sonication three drops of the dispersion were spin coated onto microscope cover slips at 3000 rpm for 20 s each. These samples were used to obtain AFM length distribution measurements.

### Spray deposition and sheet resistance measurements

Diluted AgNW dispersions were deposited on glass microscope slides using a consumer airbrush with a commercial air compressor (Impax IM200-12) set to a back pressure of 5 bar. Parallel bar electrodes were added to the substrates using silver paint (Agar Scientific G3790) and two-point resistance measurements taken using a Keithley 2400 Sourcemeter.

### UV-Visible spectrophotometry

UV-Visible spectrophotometry measurements were made using both a Campsec M350 dual beam spectrophotometer and a Shimadzu UV2501PC dual beam spectrophotometer. Measurements were taken of each substrate prior to film deposition.

### Atomic force microscopy

AFM measurements were performed on an NT-MDT microscope in semi-contact mode using tips with a resonant frequency between 200 and 300 kHz. For length distribution measurements, three 100 × 100 μm fields were taken for each sample, with the AgNW lengths measured using image analysis software (ImageJ^16^).

## Additional Information

**How to cite this article**: Large, M. J. *et al.* Predicting the optoelectronic properties of nanowire films based on control of length polydispersity. *Sci. Rep.* **6**, 25365; doi: 10.1038/srep25365 (2016).

## Supplementary Material

Supplementary Information
