# Supplementary material for: Predicting the optoelectronic properties of nanowire films based on control of length polydispersity
Source: Sci Rep. 2016 May 9;6:25365. doi: 10.1038/srep25365 (PMC4860714; doi:10.1038/srep25365)
Supplement: Supplementary Information [file srep25365-s1.pdf]

Predicting the optoelectronic performance of nanowire films based on control of length polydispersity

Matthew J. Large, Jake Burn, Alice A. K. King, Sean Ogilvie, Izabela Jurewicz, Alan B. Dalton**

University of Surrey, Guildford, Surrey, GU2 7XH, United Kingdom

*email: m.large@surrey.ac.uk; a.dalton@surrey.ac.uk

Supplementary Information

Appendix A1

First we consider the relationship between the area fraction ϕ_s and filling factor η_s. By the definition of these quantities for rod-like objects [1];

$$\eta_s = \frac{1}{L^2} \sum_{i=1}^{N} l_i^2 = \frac{\langle l^2 \rangle N}{L^2} \quad \text{(A1)}$$

$$\phi_s = \frac{1}{L^2} \sum_{i=1}^{N} l_i d_i = \frac{\langle ld \rangle N}{L^2} \approx \frac{d \langle l \rangle N}{L^2} \quad \text{(A2)}$$

where L^2 is the area of the (two-dimensional) domain; N is the total number of nanowires; l_i and d_i are the length and diameter of the ith nanowire respectively. The approximation in the latter expression assumes that the diameter of a population of wires is nominally constant; the study by [2] suggests that d and l can at least be treated as statistically independent (hence $\langle dl \rangle \approx \langle d \rangle \langle l \rangle$ where we will denote $\langle d \rangle$ by d for ease).

Inspection of equations (A1) and (A2) above yields $\phi_s = d\langle l\rangle\eta_s/\langle l^2\rangle = d\langle l\rangle n_s$, where $n_s = N/L^2$ is the number density of wires. The use of the filling factor η_s is convenient as it has a universal value for the continuum percolation threshold of isotropically distributed rods; $\eta_{s,c} = 5.6372 \ldots$ [1]. Hence we see that $n_{s,c} \approx 5.64/\langle l^2\rangle$, directly confirming the proposition of [2] and consistent with the experimental data in that study.

There is another measure of nanowire density when considering a 2D domain referred to as the *area coverage*, A_C by [3]. This is described by [1] as the "total fraction of the plane covered by objects" (which is distinct from the total area of objects per unit area of the plane due to the occurrence of overlaps), and is related to ϕ_s by;

$$A_C = 1 - e^{-\phi_s}. \quad \text{(A3)}$$

Next we take the Beer-Lambert law $-\log_{10} T = A = \varepsilon c x$ as a model for the film transmission; where $T \in [0,1]$ is the film transmittance, $\varepsilon\,[\mathrm{m^2 \cdot mol^{-1}}]$ is the molar extinction coefficient, $c\,[\mathrm{mol \cdot m^{-3}}]$ is the molar concentration, $x\,[\mathrm{m}]$ is the path length. By elimination of Avogadro's constant from ε and c, this can also be interpreted in a per-particle manner, with an extinction cross-section per particle σ and a particle number density n_v. Typically, nanowire extinction cross-sections are given per unit length (which we will term $\sigma_{ext} = \sigma_i/l_i$), so we must consider the total nanowire length per unit volume of the film. Taking the film area L^2 and path length equal to the film thickness t;

$$\sigma n_v x = \sigma_{ext} \cdot \frac{t}{L^2 t}\sum_{i=1}^{N} l_i = \frac{\sigma_{ext}}{d}\,\phi_s \quad \text{(A4)}$$

Hence we have that;

$$T = 10^{-Q_{ext}\phi_s} \quad \text{(A5)}$$

where $Q_{ext} = \sigma_{ext}/d$ is the dimensionless extinction efficiency of the nanowires.

As an aside, considering the expansion of equation (A5) to first order in ϕ_s we find that $T \approx 1 - \ln(10)\,Q_{ext}\phi_s$. From equation (A3) it is evident that for small values of ϕ_s, $A_C \approx \phi_s$, and so we can suggest that $T \approx 1 - \ln(10)\,Q_{ext}A_C$. This is directly comparable to the

empirical expression $T/\% = 100 - a_1 A_C$ given by Bergin et al. [3], and we can suggest that the fitting constant $a_1 = 100\ln(10)\,Q_{ext}$. FDTD calculations at 550 nm wavelength for nanowires of 42 nm diameter on a semi-infinite glass substrate yield $Q_{ext} = 0.37 \pm 0.04$, and hence $a_1 = 85 \pm 9$ which compares very favorably to the measured value of $a_1 = 87$ given by [3], as illustrated in figure A1.

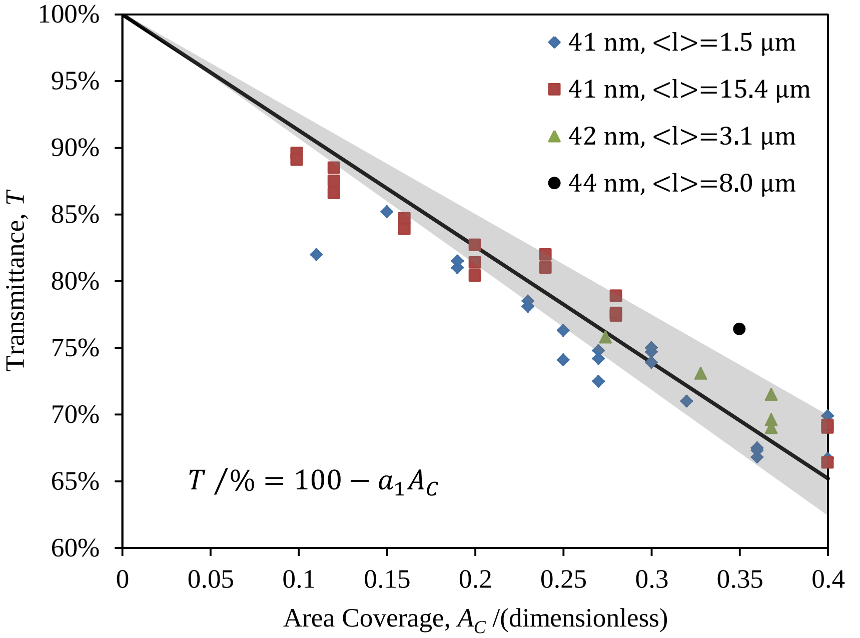

Figure A1: Modified version of Figure 4(A) from [3], showing the relationship between film transmittance and area coverage for multiple nanowire samples of different lengths but approximately constant diameters (see [3] for full details of the materials and methods used). The black line shows the inset relationship plotted with $a_1 = 87$ (from [3]) and the grey shaded area shows the bounds of an approximation based on the present theory which yields $a_1 = 100\ln(10)\,Q_{ext} = 85 \pm 9$ (based on FDTD calculations of Q_{ext}). The shaded region encapsulates approximately 60% of the data.

We can link the area fraction ϕ_s directly to the sheet resistance R_S of a nanowire film by considering the two-dimensional percolative scaling relation $\sigma_{DC} = \sigma_{DC,0}\left(\eta_s - \eta_{s,c}\right)^m$, where

σ_{DC} is the film electrical conductivity ($\sigma_{DC,0}$ is the conductivity at a filling factor of $\eta_s = \eta_{s,c} + 1$); m is the percolative scaling exponent, with suggested universal value of $m = 1.30$ for two-dimensional systems. Since we have defined a square domain $\sigma_{DC} = 1/R_S$ (with $\sigma_{DC,0} = 1/M'$ a material constant, using the notation of [2]). Utilising equations (A1) and (A2) we can invert the scaling relation to find;

$$\phi_s = \frac{d\langle l\rangle}{\langle l^2\rangle}\left[\left(\frac{M'}{R_S}\right)^{\frac{1}{m}} + \eta_{s,c}\right] \quad \text{(A6)}$$

Direct substitution of equation (A6) into (A5) gives a new T-R expression, which appropriately describes the impact of nanowire length statistics;

$$T = 10^{-Q_{ext}\frac{d\langle l\rangle}{\langle l^2\rangle}\left[\left(\frac{M'}{R_S}\right)^{\frac{1}{m}} + \eta_{s,c}\right]} \quad \text{(A7)}$$

In order to utilize equation (A7) as a fitting function for transmittance-sheet resistance data only d (and an associated calculation of Q_{ext}) need to be known; the terms M', m and $\langle l^2\rangle/\langle l\rangle$ may be used as fitting parameters (as demonstrated in the main text).

Considering the limit of equation (A7) as $R_S \to \infty$, we find that a limiting transmittance is reached; this represents the finite optical extinction of material not contributing to conduction at the percolation threshold, and is a feature which distinguishes this model from the model due to [4] where $T \to 1$ as $R_S \to \infty$. As we take the limit $R_S \to \infty$ we are left with a set of constants, which we can collect into a 'critical transmittance' term;

$$T_c = 10^{-Q_{ext}\frac{d\langle l\rangle}{\langle l^2\rangle}\eta_{s,c}} \quad \text{(A8)}$$

In the opposing limit, as R_S becomes small such that $(M'/R_S)^{1/m} \gg \eta_{s,c}$, we can show through comparison of Taylor expansions that this model is functionally equivalent to the established model of [4] (to first order);

$$T = 10^{-Q_{ext}\frac{d\langle l\rangle}{\langle l^2\rangle}\left(\frac{M'}{R_S}\right)^{\frac{1}{m}}}$$

$$\approx 1 - \ln(10)\, Q_{ext} \frac{d\langle l \rangle}{\langle l^2 \rangle} \left(\frac{M'}{Z_0}\right)^{\frac{1}{m}} \left(\frac{Z_0}{R_S}\right)^{\frac{1}{m}}$$

(A9)

Also, from [4];

$$T = \left[1 + \frac{1}{\Pi}\left(\frac{Z_0}{R_S}\right)^{\frac{1}{1+n}}\right]^{-2}$$

$$\approx 1 - \frac{2}{\Pi}\left(\frac{Z_0}{R_S}\right)^{\frac{1}{1+n}}$$

(A10)

By equating the coefficients of terms in $(Z_0/R_S)^{1/m}$, we can suggest a figure of merit Π';

$$\Pi' = \frac{2\left(\frac{Z_0}{M'}\right)^{\frac{1}{m}} \langle l^2 \rangle}{\ln(10) Q_{ext} d\langle l \rangle} \quad \text{(A11)}$$

which is described by parameters of the percolation scaling, parameters of the nanowire length distribution and their optical interactions (as well as the physical constant Z_0, the impedance of free space). In the case that $m = 1 + n, \Pi' = \Pi$.

Acknowledgement

The authors would like to gratefully acknowledge funding from M-Solv Ltd and the UK Defense Science and Technology Laboratory (DSTL).

References

1. Mertens, S.; Moore, C., Continuum percolation thresholds in two dimensions. Phys Rev E 2012, 86 (6), 061109.

2. Khanarian, G.; Joo, J.; Liu, X. Q.; Eastman, P.; Werner, D.; O'Connell, K.; Trefonas, P., The optical and electrical properties of silver nanowire mesh films. J Appl Phys 2013, 114 (2), 024302.

3. Bergin, S. M.; Chen, Y. H.; Rathmell, A. R.; Charbonneau, P.; Li, Z. Y.; Wiley, B. J., The effect of nanowire length and diameter on the properties of transparent, conducting nanowire films. Nanoscale 2012, 4 (6), 1996-2004.

4. De, S.; King, P. J.; Lyons, P. E.; Khan, U.; Coleman, J. N., Size Effects and the Problem with Percolation in Nanostructured Transparent Conductors. ACS Nano 2010, 4 (12), 7064-7072.
